# The Impact of Recent Rubella Vaccine Introduction in 5 Countries in The African Region

**Published:** 2018-07-28

**Authors:** Richard Luce, Balcha G Masresha, Regis Katsande, Amadou Fall, Messeret Eshetu Shibeshi

**Affiliations:** 1 WHO Inter-country Support Team for Central Africa, Libreville, Gabon; 2 WHO Regional Office for Africa, Brazzaville, Congo; 3 WHO Inter-country Support Team for Western Africa, Ouagadougou, Burkina Faso; 4 WHO Inter-country Support Team for East and Southern Africa, Harare, Zimbabwe

**Keywords:** Impact, New Vaccine Introduction, Rubella, Vaccines, Vaccine Preventable Diseases

## Abstract

The World Health Organization (WHO) recommends that countries introduce rubella containing vaccines (RCVs) to reduce rubella circulation and the occurrence of congenital rubella syndrome (CRS). As of June 2017, a total of 18 countries have already introduced or are in the process of introducing RCV in routine child vaccination programs. RCV introduction during 2013 - 2014 in five countries in the Region resulted in a reduction of rubella incidence of 48% to 96% in the post-introduction period as compared to the average incidence in the years before introduction. These results suggest that initial mass vaccination campaigns and introduction of RCVs in routine immunization programs result in significant reduction in rubella incidence and a reduced potential for the occurrence of CRS.

## Introduction

Rubella virus infection is a disease of usually mild symptomology affecting primarily children; however, infection of women during early pregnancy can result in congenital rubella syndrome (CRS) causing deafness, blindness, congenital heart disease and mental retardation in children. In 2010, more than 100,000 babies with congenital rubella syndrome (CRS) were estimated to be born globally^1^. Effective vaccines exist to prevent rubella infection and to reduce CRS, which is the main objective of rubella vaccination. Rubella and CRS elimination was achieved in September 2016 in the Americas^2^,^3^. The same objective has been formally adopted by WHO regions of the world except the Eastern Mediterranean and African Regions^4^,^5^.

WHO recommends that countries use vaccination campaigns as a means of initially introducing rubella containing vaccines before replacing monovalent measles vaccine with measles-rubella (MR) vaccine in the routine immunization schedule. In order to prevent displacement of rubella susceptibility and circulation among older age groups and pregnant women, thereby increasing the potential for increasing CRS cases, WHO further recommends that countries achieve 80% measles coverage in routine vaccination and/ or in SIAs before MR introduction^6^.

Previous studies of rubella transmission in the African region among women of child-bearing age, have documented widespread circulation of rubella virus^7^–^10^. Analysis of measles surveillance data in the African region, which makes rubella testing on measles IgM negative specimens, found that 95% of rubella IgM positive cases occurred in children ≤ 15 years of age in the years between 2002 and 2009^11^. Thus, the occurrence of approximately 5% of cases in persons older than 15 years of age suggests a risk of CRS occurrence among women of child bearing age. Sentinel CRS surveillance is operational in 6 countries in the African region and CRS cases have been documented^12^.

Cape Verde, Mauritius and the Seychelles introduced MR vaccine prior to 2010 and were the first countries in the region to vaccinate against rubella. As of June 2017, 15 other countries have conducted mass vaccination campaigns using MR vaccine prior to its introduction in their childhood immunization programs: Botswana, Burkina Faso, Cameroon, Cape Verde, Gambia, Ghana, Kenya, Namibia, Rwanda, São Tomé and Principe, Senegal, Swaziland, Tanzania, Zambia and Zimbabwe^13^. This report analyzes rubella surveillance data from the first 5 countries to introduce MR in the WHO Africa Region during 2013-14 by comparing incidence in the pre and post introduction period.

## Methods

We analyzed case-based surveillance data for the 10 year period from 2007 to 2016 in 5 countries that introduced MR vaccine in 2013 and 2014: Burkina Faso, Ghana, Rwanda, Senegal and Tanzania. Specific rubella surveillance systems do not exist in these countries. Data were collected through the national measles surveillance systems that use a standardized WHO regional case definition of fever and generalized maculopapular rash, and at least 1 of the following symptoms: cough, coryza (runny nose), or conjunctivitis. Each suspected case is reported on a case investigation form to collect information on age, sex, vaccination status and district of residence. Blood specimens are collected from 5 suspect cases when there is suspicion of an outbreak. Specimens that test negative or indeterminate for measles-specific immunoglobulin M (IgM) antibody are tested for the presence of rubella-specific IgM antibody using a standard enzyme-linked immunosorbent assay. A confirmed rubella case is defined as an IgM negative measles cases that is IgM positive for rubella. National measles/rubella laboratories are regularly accredited according to performance on specific criteria by WHO AFRO^14^.

The incidence rate of rubella was calculated by dividing the number of confirmed rubella cases reported in a year by the total population for the calendar year and expressed as a rate per million population. The pre-introduction period was the years before the introduction of rubella vaccine and the year of the initial MR SIAs. The post-introduction period was the years following the initial MR catch-up SIAs. The mean annual number of rubella cases was calculated by taking the number of rubella cases reported during the specific period of years in consideration divided by the number of years.

Surveillance performance is measured using standard indicators, of which two are considered principal monitoring indicators^a^. The non-measles febrile rash illness rate measures the level of case finding and investigation taking place in countries. The proportion of districts investigating suspected measles cases with blood specimen attempts to measure the representativeness of all subnational administrative units in the case investigation efforts. The targets for performance are as follows: a non-measles febrile rash illness rate of at least 2 per 100,000; at least 80% districts reporting suspected measles cases with a blood specimen per year.

## Results

**Figure 1:**
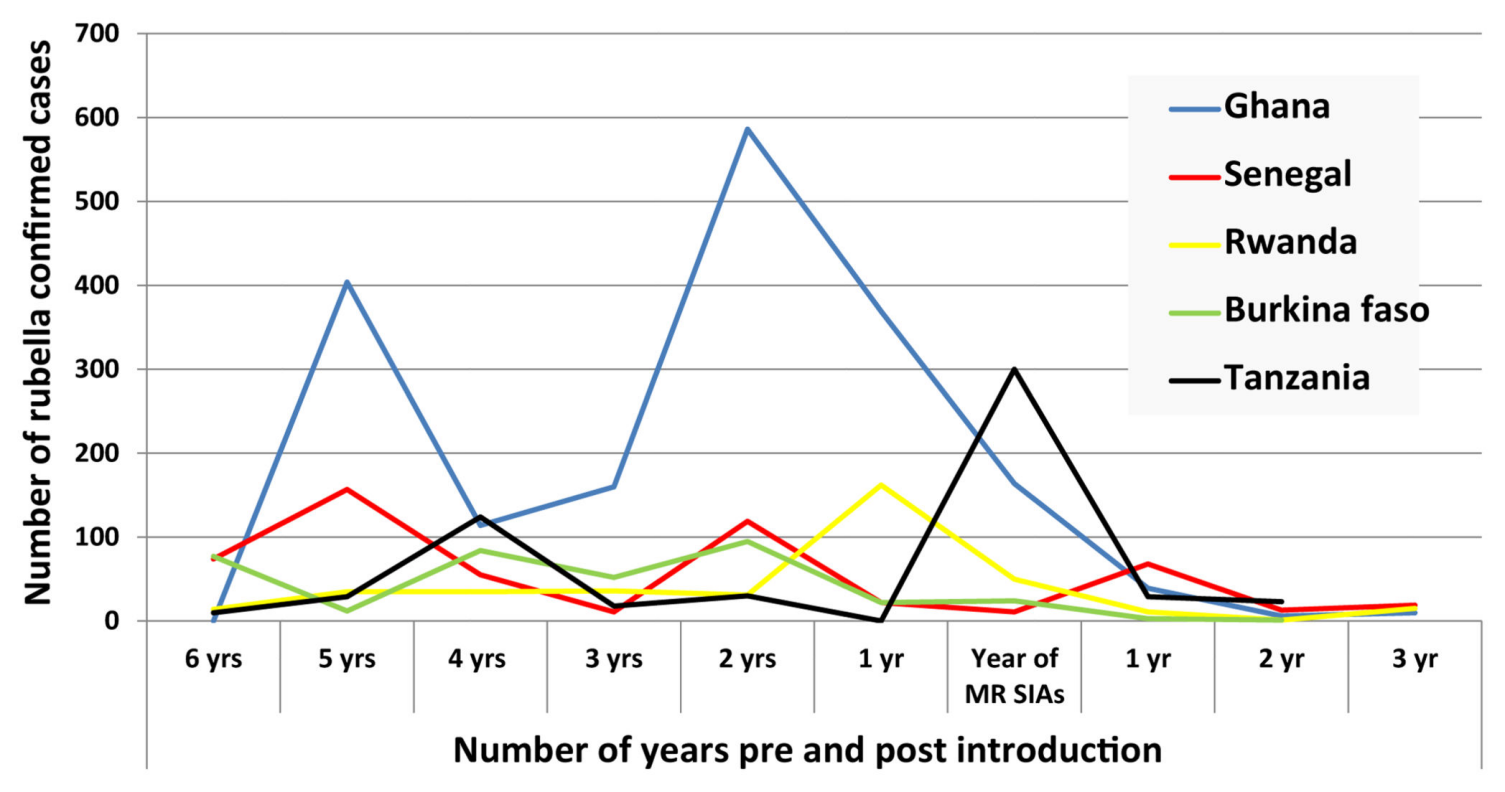
Lab confirmed rubella cases before and after MR SIAs in the five countries. 2007 - 2016.

All five countries introduced MR by mass vaccination campaign during 2013-2014 targeting children from 9 months to 14 years of age before introducing the vaccine in the routine immunization schedule. In each country, rubella cases were confirmed by laboratory testing in almost each year; however, the number of confirmed cases varied by year and by country. As many as 586 cases were reported from Ghana in 2011 prior to MR vaccination and a low of 1 case each in Rwanda in 2015, and in Burkina Faso in 2016, after MR vaccination commenced. Ghana reported 1852 confirmed cases, which was the highest cumulative number over the ten-year period, while Burkina Faso reported a total of 373 confirmed cases, which was the lowest cumulative number of cases among the five countries. Figure 1 shows the annual number of cases of confirmed rubella in the years before and following the MR SIAs.

In all five countries, the mean annual number of confirmed cases decline ranged from 48% to 96% when comparing the pre-introduction and post-introduction periods. Mean annual confirmed rubella incidence also declined in all five countries when comparing the pre- and post-campaign periods. Ghana experienced the largest percentage decline in mean annual confirmed rubella incidence of 81%. The other countries experienced incidence declines of 31% to 57% (Table 1). Tanzania conducted its MR catch-up SIAs in October 2014. The country had experienced rubella outbreaks between March and September 2014, just preceding the MR SIAs.

The quality of measles-rubella surveillance in these 5 countries is indicated in Table 2. Burkina Faso and Tanzania had failed to meet the targets for the principal performance indicators in some of the years. The other three countries have achieved good standard performance in at least 7 of the most recent years.

The number of children vaccinated and administrative coverage result from the MR catch-up SIAs are shown in table 3. Administrative coverage data from the MR mass vaccination campaigns was high; greater than 100% in all countries except Ghana where it was 99%.

The proportion of cases among children under 5 years of age was highest in Senegal at 37.5% and lowest in Burkina Faso at 19%. The proportion of cases over 15 years of age varied from a low of 2.1% in Rwanda to a high of 10.5% in Burkina Faso (Table 4). The proportion of confirmed cases in the over 15 years age group declined in the post-introduction period from 5.3% to 3.8% in Tanzania, from 9.0% to 7.3% in Ghana and from 2.2% to 1.3% in Rwanda. This proportion increased slightly in Senegal from 2.2% to 4.0% of cases.

## Discussion

Rubella virus circulates widely in Africa, predominantly in children under 15, with about 5% of cases occurring in those over 15 years of age^5^. Other studies from African countries have also documented that rubella infections occur in the childhood age group prior to the introduction of the rubella containing vaccine^15^, ^16^, ^17^,^18^.

The five countries in this study were among the first in the African region to introduce MR vaccine through a mass vaccination campaign followed by introduction into the routine childhood vaccination system. Large reductions in rubella transmission were identified in each country following the pattern described after implementation of mass vaccination campaigns conducted as part of rubella elimination efforts in the Americas^19^. During the period from 1997 to 2008, the countries of the Americas introduced RCVs by organizing 3 initial mass campaigns. In general, the strategy involved 2 campaigns targeting children 1-4 years of age and the third targeting adolescent and adults. The intervals between the campaigns ranged from 2 to 6 years and the sequence of age group targeted was adapted to local epidemiological considerations. From 1998 to 1996, the number of rubella cases declined by 98% in the region of the Americas^3^. In Brazil, seronegativity for rubella declined from 9.4% before a nationwide rubella immunization campaign to 2.8% in the post-campaign period^20^. Similarly, a significant increase in seropositivity for rubella was observed after an MR catch-up campaign in Iran^21^. In contrast, these initial MR introductory campaigns in Africa targeted children from 9 months to 14 years of age and the observed reduction in the number of rubella cases varied from 48%-98%.

As with measles elimination strategies, high routine immunization coverage in the childhood vaccination program and periodic follow-up campaigns using MR vaccine are necessary to maintain population immunity against rubella infection and sustain reduced viral circulation.

Surveillance for CRS is not conducted in all countries in the African Region; however, estimates of CRS incidence prior to RCV introduction suggest that countries in Africa have rates between < 50 cases to more than 150 cases per 100 000 lives births; with a majority of countries in the range of 100-150 cases per 100 000 live births^1^. CRS sentinel surveillance is operational in a limited number of countries in the region including Zimbabwe, Tanzania, Togo, Uganda, Rwanda, Burkina Faso, Senegal, and Zambia. A cumulative total of 267 suspected cases were reported in these countries with 103 meeting the case definition of confirmed CRS in 2015^12^. Rwanda, Uganda and Swaziland conducted retrospective record reviews for CRS cases at multiple pediatric hospitals in each country over 2 to 4 year periods up until 2012. Cases clinically compatible with CRS were identified in each country; 51 cases in Rwanda, 22 in Uganda and 5 in Swaziland^22^. Similar findings were reported in Morocco^22^. The establishment of additional functional CRS sentinel surveillance sites is necessary to better analyze the trend of CRS cases and demonstrate the impact of future MR introductions.

The results of this analysis demonstrate that the incidence of rubella cases can be reduced significantly in the years immediately after mass vaccination campaigns; however, a single campaign followed by MR introduction in routine vaccination with coverage below 95% may not be adequate to maintain population immunity without additional follow-up MR mass campaigns. Active surveillance for febrile rash illnesses, and CRS sentinel surveillance need to be expanded and strengthened in order to demonstrate the impact of MR vaccination and fully describe the impact on disease burden.

## Limitations

Surveillance data was available for 2 – 3 years following the introduction of rubella vaccine in the respective countries and may not be indicative of longer term trends which depend on the vaccine coverage in routine vaccination and in future mass campaigns. The quality of surveillance determines the ability of countries to identify and investigate suspected cases of fever and rash. Even though rubella testing is done on specimens testing negative for measles IgM antibodies, the surveillance systems used by the countries uses the case definition for suspected measles cases, which may not capture rubella cases presenting with only fever and rash that do not exhibit cough, coryza or conjunctivitis.

## Figures and Tables

**Table 1:** Mean annual IgM positive rubella cases by country before and after the MR campaign. 2007 – 2016.

Country	Mean annual number of confirmed rubella cases			Mean incidence of confirmed rubella cases (per million population)		
	pre-MR SIAs	post-MR SIAs	% change	pre-MR SIAs	post-MR SIAs	% change
**Burkina Faso**	46	2	-96%	4.9	0.8	-84%
**Ghana**	257	18	-93%	5.1	2.2	-57%
**Rwanda**	52	9	-83%	10.4	0.7	-93%
**Senegal**	64	33	-48%	74.4	2.9	-96%
**Tanzan**ia	65	26	-60%	1.3	0.5	-62%

**Table 2:** Measles surveillance performance in the 5 countries according to the two principal performance indicators. 2007 - 2016.

		2007	2008	2009	2010	2011	2012	2013	2014	2015	2016
		
% districts reporting	Burkina Faso	65%	95%	96%	95%	98%	100%	90%	95%	77%	79%
	Ghana	93%	91%	96%	96%	96%	100%	100%	100%	100%	100%
	Rwanda	85%	64%	72%	100%	100%	100%	100%	94%	94%	94%
	Senegal	91%	78%	86%	86%	85%	100%	100%	89%	100%	91%
	Tanzania	67%	32%	60%	94%	75%	94%	81%	100%	99%	100%
	
non-measles febrile rash illness rate per 100,000 population	Burkina Faso	0.8	1.4	0.9	2.3	1.4	3.3	1.9	2	0.6	0.8
	Ghana	0	4.1	2.1	2.7	6.3	4.8	2.8	3.3	3.5	2.6
	Rwanda	1.2	1.8	2.6	4	3.2	6.5	6.4	3.7	3.6	7.3
	Senegal	3.2	3.9	3.8	3.2	2.7	2.2	3.8	6.8	3.6	5.1
	Tanzania	1.4	0.2	0.8	1.9	0.6	1.2	0.1	1.7	0.8	1.9
	

**Table 3:** Administrative coverage and mean incidence of lab confirmed rubella in the pre and post SIAs period. 2007 – 2016.

Country	Year of MR SIAs	MR SIAs target population	MR SIAs national administrative coverage
**Rwanda**	12 - 15 March 2013	4,391,081	103%
**Ghana**	11 - 20 Sept 2013	11,062,605	99%
**Senegal**	18 - 27 Nov 2013	6,097,123	101%
**Burkina Faso**	21 – 30 Nov 2014	8,517,508	107%
**Tanzania**	18 - 24 Oct 2014	8,168,395	101%

**Table 4:** Age breakdown of lab confirmed (IgM positive) rubella cases by country. 2007-2016.

Age group	Burkina Faso	Ghana	Rwanda	Senegal	Tanzania
**< 5 years**	19.0%	35.0%	34.4%	37.5%	32.7%
**5 to 9 years**	46.6%	36.8%	49.7%	48.6%	38.5%
**10 to 14 years**	23.9%	19.4%	13.8%	11.3%	23.7%
**> 15 years and above**	10.5%	8.9%	2.1%	2.6%	5.1%
**Total**	100.0%	100.0%	100.0%	100.0%	100.0%

## Footnotes

a The non-measles febrile rash illness rate, and the proportion of districts investigating suspected measles cases with blood specimen.
